# Transforming cervical cancer pathological diagnosis through artificial intelligence: progress, performance, and barriers to clinical implementation

**DOI:** 10.3389/fonc.2025.1716018

**Published:** 2026-01-20

**Authors:** Yue Zhang, Jiangbo Yuan, Lin Chen

**Affiliations:** 1 Shaanxi Provincial People’s Hospital, Xi’an, China; 2 Pucheng County Hospital, Weinan, China

**Keywords:** artificial intelligence, cervical cancer, pathological diagnosis, deep learning, machine learning, digital pathology

## Abstract

**Objective:**

Cervical cancer faces significant pathological diagnosis challenges including pathologist shortages, subjective interpretation, and inconsistent detection rates. This systematic review evaluates AI technology’s application status, development level, and key challenges in cervical cancer pathological diagnosis.

**Methods:**

A systematic literature review across three databases (PubMed/MEDLINE, Scopus, Web of Science) covering January 2015 to August 2025. Search terms included “artificial intelligence,” “cervical cancer,” “pathological diagnosis,” “histopathology,” “machine learning,” and “deep learning.” Studies involving AI applications in cervical cancer pathological diagnosis were included, encompassing histopathological, immunohistochemical, and molecular pathological diagnoses. Animal studies, cytological screening, and genomic analyses unrelated to pathological diagnosis were excluded.

**Results:**

From 1,847 identified articles, 56 studies were included. AI technology demonstrated substantial potential in histopathological image analysis, diagnostic support systems, and accuracy validation. Deep learning architectures, particularly convolutional neural networks, achieved 92-98% diagnostic accuracy while reducing processing time from 8–15 minutes to 1–3 minutes per case. However, significant implementation challenges persist including standardization issues, limited clinical validation, and substantial infrastructure costs.

**Conclusion:**

AI technology shows broad application prospects in cervical cancer pathological diagnosis, potentially alleviating pathologist shortages and improving diagnostic standardization. The technology particularly suits cervical cancer prevention in resource-limited regions, supporting global elimination goals, though standardization and validation challenges require addressing before widespread clinical implementation.

## 1 Introduction

Cervical cancer remains the fourth most common cancer among women globally and represents the fourth leading cause of cancer-related deaths, with approximately 570,000 new cases and 310,000 deaths occurring worldwide annually (1). This malignancy demonstrates pronounced geographical disparities, serving as the most prevalent cancer in 23 countries and the leading cause of cancer mortality in 36 nations, predominantly located in sub-Saharan Africa, Melanesia, South America, and Southeast Asia (2). Despite being largely preventable due to its prolonged precancerous phase (3), cervical cancer continues to pose significant challenges to global health systems (4). Pathological diagnosis serves as the gold standard for cervical cancer detection, playing a crucial role in disease confirmation, grading, staging (5), and treatment decision-making (6). However, traditional pathological diagnostic approaches face substantial limitations, particularly the critical shortage of qualified pathologists worldwide, especially in developing countries where the pathologist-to-population ratio remains severely inadequate (7). Manual screening approaches are not always accurate and may result in certain lesions remaining undetected for extended periods, highlighting the inherent limitations of conventional diagnostic methods (8). The diagnostic process heavily relies on subjective interpretation by pathologists, leading to inter-observer and intra-observer variability that can compromise diagnostic consistency and reproducibility (9).

The emergence of artificial intelligence (AI) has demonstrated remarkable potential in addressing these pathological diagnostic challenges through automated analysis capabilities (10). Recent advances in digital pathology technology have established a solid foundation for AI applications in pathological diagnosis (11), with whole-slide imaging technology enabling high-resolution digital storage, transmission, and analysis of pathological specimens (12). AI has increasingly been utilized for diagnosing various diseases, including skin cancer classification (13), tumor imaging diagnosis (14), retinal disease detection (15), and gynecological cancers (16). Through sophisticated algorithms, AI systems can autonomously identify images, learn classifications, extract features, and process data with remarkable precision. The breakthrough in deep learning technology, particularly the successful application of convolutional neural networks in medical image analysis, has enabled AI systems to automatically learn complex morphological feature patterns from large pathological image datasets, achieving high-precision automated diagnosis. This capability not only reduces the workload burden on pathologists but also holds promise for improving diagnostic standardization and consistency.

While AI has demonstrated advances in cervical cytology screening (Pap smear and liquid-based cytology) (9), this review focuses specifically on AI applications in histopathological diagnosis of cervical tissue specimens. Histopathological examination remains the definitive gold standard for confirming cervical cancer diagnosis and guiding treatment decisions (5). To maintain methodological consistency and provide focused insights, we excluded purely cytological screening studies. This approach allows comprehensive evaluation of AI technologies specifically designed for tissue-based pathological diagnosis, including histopathological, immunohistochemical, and molecular pathological applications.

Despite growing evidence supporting AI-assisted technology for histomorphological analysis and cervical epithelial dysplasia identification, comprehensive evaluation of AI performance and challenges in cervical cancer pathological diagnosis requires systematic investigation. While most AI-supported pathology technologies remain in developmental or observational research phases (17), their implementation in routine clinical practice is accelerating (18, 19). This study aims to systematically review the development trajectory, technological breakthroughs, and clinical application outcomes of AI technology in cervical cancer pathological diagnosis. Through comprehensive analysis of current technical challenges, clinical translation barriers, and regulatory policy issues, this research will provide theoretical foundations and practical guidance for advancing AI technology implementation in cervical cancer diagnosis and treatment. The findings will contribute to enhanced global cervical cancer prevention and control efforts, particularly in resource-limited regions where healthcare disparities remain most pronounced.

## 2 Materials and methods

A systematic review of published articles on the development or validation of artificial intelligence techniques for pathological diagnosis of cervical cancer is presented.

### 2.1 Research questions

This review is based on the following research questions: How much do we know about the application of AI in cervical cancer pathological diagnosis, how accurate and how far has AI technology developed in cervical cancer pathological diagnosis, and what are the key challenges?

### 2.2 Search strategies and information sources

This study was designed as a systematic review. We conducted literature searches in three databases: PubMed/MEDLINE, Scopus, and Web of Science, covering the period from January 2015 to August 2025. The search keywords included “artificial intelligence” combined with terms such as “cervical cancer”, “pathological diagnosis”, “histopathology”, “digital pathology”, “immunohistochemistry”, “molecular pathology”, “cervical intraepithelial neoplasia (CIN)”, “machine learning”, “deep learning”, “convolutional neural network”, “whole slide imaging”, “computer-aided diagnosis”, “detection”, and “diagnosis”. Additionally, MeSH keywords and Boolean operators (AND, OR) were employed to enhance the screening of search results.

### 2.3 Inclusion and exclusion criteria

We included all types of studies involving pathological diagnosis of cervical cancer, including those with histopathological, immunohistochemical, or molecular pathology diagnoses, to comprehensively assess the application progress of AI in cervical cancer pathology. These studies were conducted worldwide and primarily published in English. Excluded studies included: animal research; purely cytological screening studies (Pap smear and liquid-based cytology without histopathological confirmation; non-histopathological diagnosis); nuclear segmentation technology studies; genomic mapping analyses; pure biomarker discovery; chromosomal variation analysis; gene expression profiling; photoelectric sensor technologies; spectroscopic studies; purely mathematical modeling; and prognostic prediction studies for cervical cancer. Additionally, we excluded research on cancer lesion segmentation in MRI/CT imaging, colposcopy image analysis, non-full-text articles, case reports, systematic reviews, Meta analyses, editorials, conference abstracts, and technical reports. The focus was on studies involving pathological section image analysis, diagnostic support, and validation of diagnostic accuracy. Poor methodology” was determined based on the following criteria: lack of clear AI model description, absence of validation dataset, sample size fewer than 50 cases, missing performance metrics (sensitivity, specificity, or accuracy), inadequate comparison with pathologist assessment, or insufficient detail for reproducibility assessment. Two independent reviewers evaluated methodological quality, with discrepancies resolved through discussion with a third reviewer.

### 2.4 Study selection

The three authors collaboratively reviewed, screened, and analyzed the retrieved literature. EndNote software (EndNote X20, Clarivate) was used to manage the literature, with inclusion criteria applied during the screening process. In case of disputes, a third expert author would serve as arbitrator. Initial screening was conducted based on the relevance of titles and abstracts, followed by full-text verification to ensure compliance with inclusion criteria. Special attention was given to AI application studies involving histopathological diagnosis, immunohistochemical diagnosis, and molecular pathology diagnosis of cervical cancer.

### 2.5 Data extraction and synthesis

All included articles underwent systematic extraction and organization using pre-designed customized data extraction forms to ensure standardized and consistent data collection. The extracted data encompassed author information, publication year, research design type, sample size, AI technology type, pathological diagnosis application fields, diagnostic performance metrics (sensitivity, specificity, accuracy), technical advantages, application challenges, clinical validation status, and key conclusions. The data extraction was independently conducted by two authors, with any disagreements resolved through thorough discussions (with third-party external experts involved when necessary). Given the diversity of research designs and reporting formats, we employed a systematic synthesis approach to integrate and analyze research findings, systematically organizing them according to the chronological progression of applications and the types of challenges encountered.

## 3 Results

### 3.1 Search results

**Figure 1:**
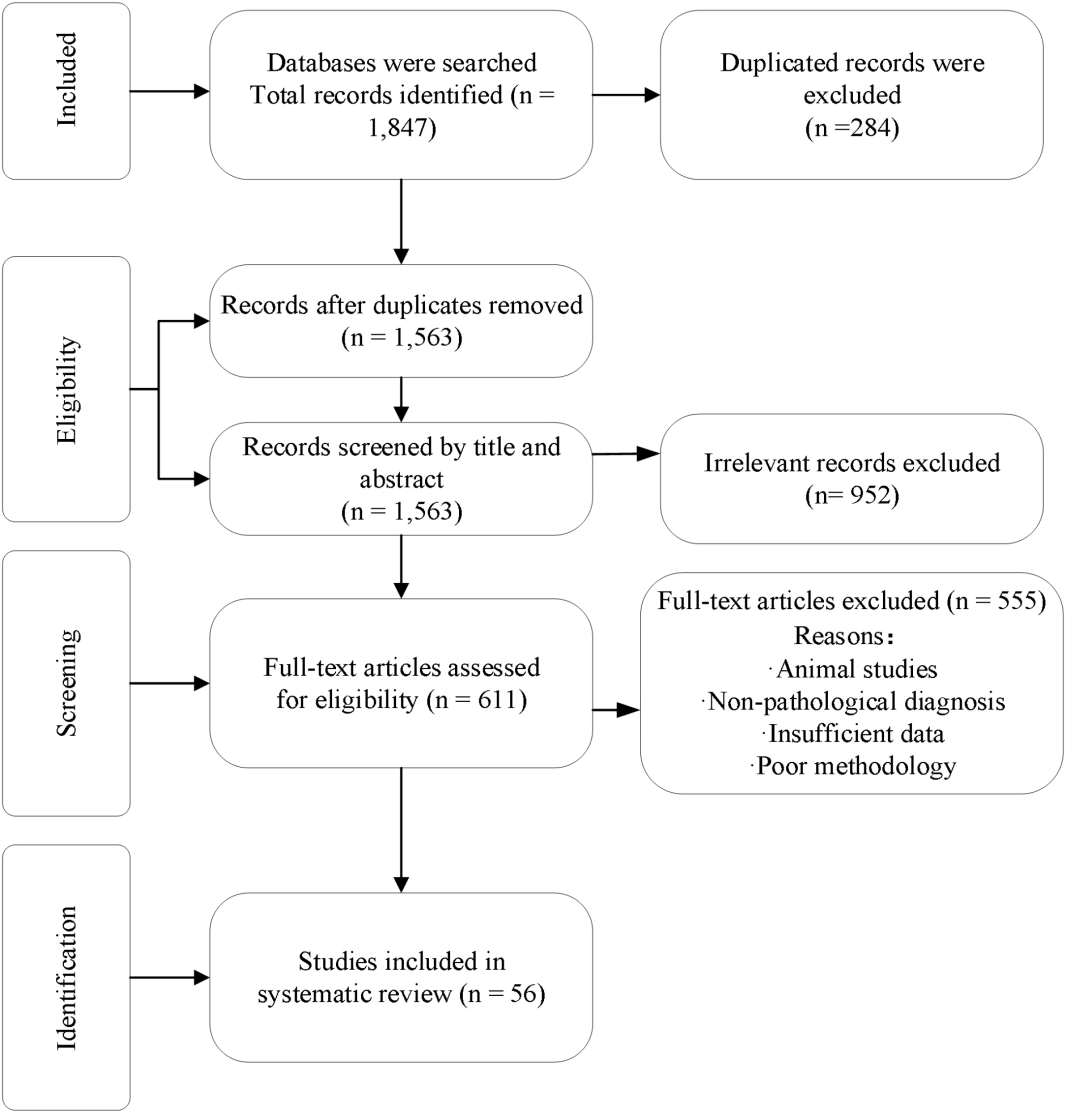
PRISMA flow diagram for systematic review study selection.

Through comprehensive searches of three databases—PubMed/MEDLINE, Scopus, and Web of Science—a total of 1,847 publications were identified, with 284 duplicates excluded. After reviewing titles and abstracts of the remaining papers, 952 were eliminated. Of the remaining articles, 555 were excluded as detailed in **Table 1**. The primary exclusion reasons included poor methodological quality (n=187, 33.7%), purely cytological screening studies (n=103, 18.6%), and non-histopathological diagnostic studies (n=94, 16.9%). Ultimately, 56 studies were included in the systematic review (**Figure 1**).

**Table 1:** Categories and numbers of full-text articles excluded (n=555).

Exclusion Category	Number of Studies	Percentage (%)
Poor methodological quality	187	33.7
Purely cytological screening studies	103	18.6
Non-histopathological diagnostic studies	94	16.9
Technical or algorithm development studies	78	14.1
Review articles and meta-analyses	42	7.6
Imaging-based studies (MRI/CT/colposcopy)	28	5.0
Conference abstracts and non-full-text articles	23	4.1
Total	555	100

The 56 included studies evaluated diverse AI approaches. Custom deep learning models were developed in 34 studies (60.7%), using ResNet (n=12), DenseNet (n=8), VGG (n=6), EfficientNet (n=4), and Vision Transformers (n=4). Fourteen studies (25.0%) employed traditional machine learning (SVM, Random Forests, KNN), and 8 studies (14.3%) investigated commercial AI-assisted diagnostic platforms. Most systems (n=43, 76.8%) functioned as AI-assisted tools requiring pathologist review, while 13 (23.2%) were designed for autonomous screening with pathologist oversight. None operated as fully autonomous replacements. Most studies (n=51, 91.1%) focused on histopathological image analysis, while 5 (8.9%) addressed immunohistochemical marker quantification.

### 3.2 AI technology evolution and cost-effectiveness challenges

The technological evolution of artificial intelligence in cervical cancer pathological diagnosis has demonstrated remarkable progression since the maturation of digital pathology technology in 2010, offering new opportunities to address the critical shortage of qualified pathologists worldwide (17, 18). Early investigations primarily employed traditional machine learning approaches, including support vector machines (SVM), random forests (RF), and k-nearest neighbors (KNN) algorithms (20). Mehmood et al. established that these foundational methods created the groundwork for AI pathological diagnosis but exhibited inherent limitations requiring manual feature engineering (21). Giansanti et al. demonstrated that diagnostic accuracies typically ranged from 70-80% in complex pathological image analysis using these conventional approaches (22). The introduction of deep learning technology after 2016 revolutionized cervical cancer pathological diagnosis (10, 11) while simultaneously introducing novel technical challenges. Sambyal et al. documented that contemporary deep learning architectures encompass convolutional neural networks (CNN), deep neural networks (DNN), residual networks (ResNet), and advanced Vision Transformer (ViT) implementations (23). Khare et al. reported that these systems demonstrate autonomous learning capabilities for complex pathological feature patterns without manual feature engineering, achieving diagnostic accuracies of 92-98% (24). However, Du et al. identified that each technological advancement presents distinct limitations: CNNs face receptive field constraints, ResNets resolve gradient vanishing issues while significantly increasing computational complexity, and ViTs achieve global feature understanding but require unprecedented volumes of annotated data (25).

**Figure 2:**
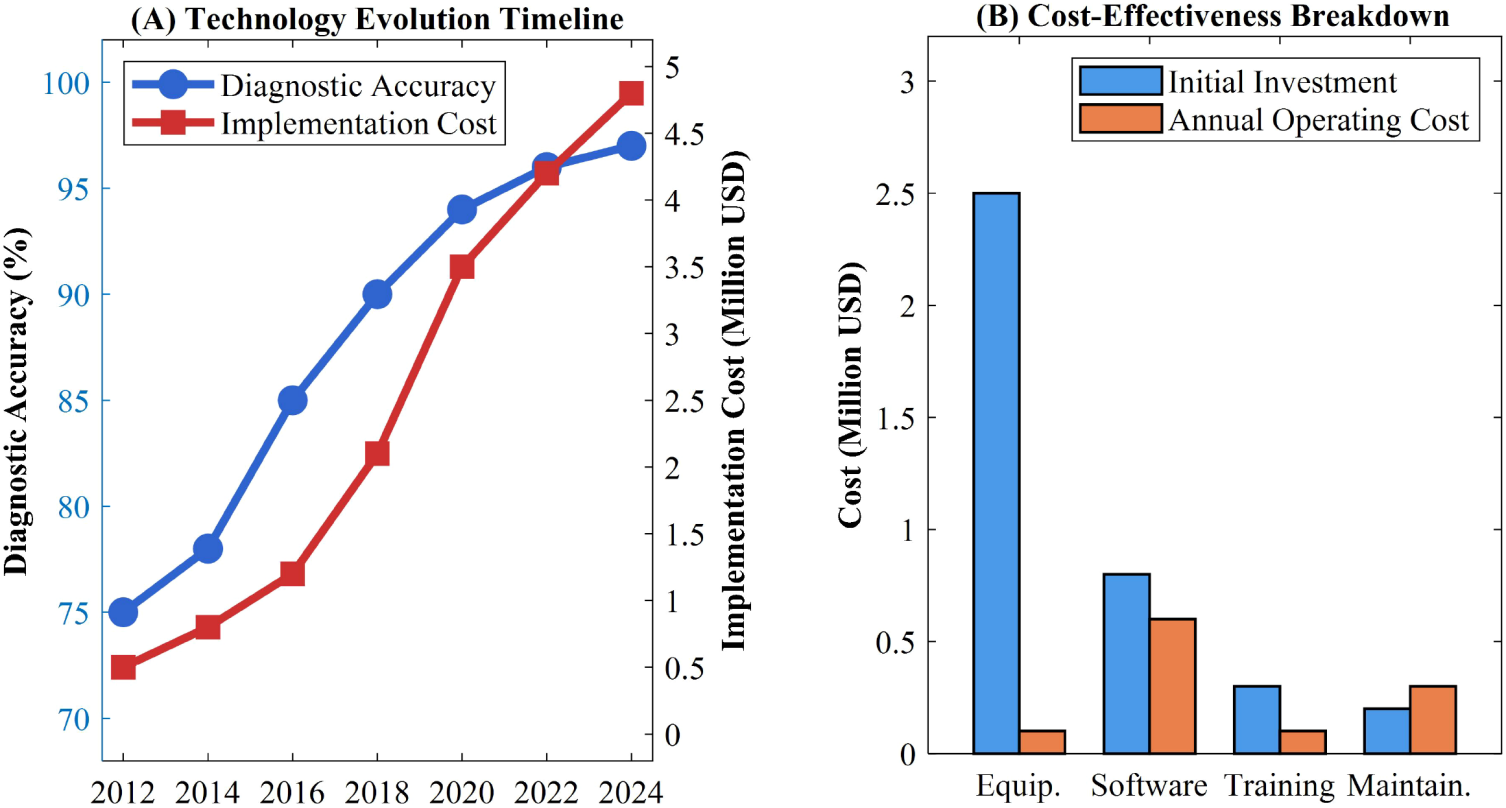
AI algorithm evolution and performance progression in cervical pathology. **(A)** Technology evolution timeline showing diagnostic accuracy and implementation cost from 2012 to 2024. **(B)** Cost-effectiveness breakdown comparing initial investment and annual operating costs across equipment, software, training, and maintenance

Recent hybrid deep learning techniques have emerged as predominant trends, incorporating generative adversarial networks (GAN) for data augmentation, attention mechanisms for critical region localization, and multi-scale fusion networks for whole slide image analysis (26). Vargas-Cardona et al. demonstrated that these systems enable processing of high-resolution whole slide images (WSI), facilitating multi-scale intelligent analysis from low-magnification overviews to high-magnification details (27). Nevertheless, Xue et al. highlighted that the exponential growth in technical complexity introduces challenges including insufficient algorithm interpretability, enormous computational resource requirements, and inconsistent model generalization capabilities (28). The cost-effectiveness analysis reveals significant dual characteristics in AI pathological diagnosis implementation. Xue et al. established that AI-assisted diagnosis substantially improves efficiency, reducing traditional pathological analysis time from 8–15 minutes to 1–3 minutes per cervical biopsy case, representing 4–6 fold efficiency improvements (29). Jeleń et al. documented substantial initial investment costs, including digital pathology scanning equipment ($200,000-500,000), annual AI software licensing fees ($50,000-150,000), and comprehensive technical personnel training (30). Bao et al. emphasized that these create formidable barriers for widespread adoption, particularly in resource-limited healthcare settings (31). Zhao et al. conducted quantitative analysis demonstrating that the technological progression shows both remarkable achievements and persistent implementation challenges (32). As shown in **Table 2** and **Figure 2**, the technological progression demonstrates both remarkable achievements and persistent implementation challenges.

**Table 2:** Evolution of AI algorithms in cervical cancer pathological diagnosis.

Algorithm type	Period	Accuracy range	Processing time	Key advantages	Main limitations	
Traditional ML (SVM, RF, KNN)	2015-2017	70-80%	2–4 hours	Simple implementation, interpretable	Manual feature engineering required
Early CNN	2017-2019	85-90%	30–60 min	Automatic feature learning	Limited receptive field, shallow features
Advanced CNN/ResNet	2019-2021	90-94%	10–20 min	Deep feature extraction, gradient optimization	High computational complexity
ViT & Attention Models	2021-2023	92-96%	5–10 min	Global feature understanding, attention mechanism	Large annotated dataset requirements
Hybrid & Multi-modal	2023-2025	94-98%	1–5 min	Multi-scale integration, ensemble learning	Algorithm interpretability challenges

The most recent advancement represents pathology-specific foundation models, demonstrating state-of-the-art performance and superior generalization. Foundation models (e.g., UNI, CHIEF, Virchow) are pretrained on massive datasets comprising millions of whole-slide images using self-supervised learning. Unlike conventional CNNs requiring task-specific training, these models learn universal pathological features from 100,000+ WSIs. Deep neural network models have demonstrated superior performance in computational histopathology applications (10). For cervical cancer diagnosis, these models show promise in handling morphological heterogeneity, distinguishing subtle dysplastic changes, and integrating multi-scale information. However, challenges persist including substantial computational requirements (GPU clusters with 40-80GB VRAM), limited interpretability, and accessibility concerns for resource-limited institutions. Foundation models fundamentally reshape AI pathology, offering standardized high-performance diagnostics, though implementation barriers remain significant (24).

### 3.3 AI predictive model performance and clinical validation challenges

Artificial intelligence-based predictive models for cervical cancer pathology have demonstrated remarkable technological breakthroughs in recent years, with deep learning architectures achieving unprecedented accuracy in diagnostic and prognostic predictions (32). Esteva et al. developed breakthrough approaches in medical image classification using deep neural networks, demonstrating the capability of end-to-end learning frameworks to automatically extract complex features from pathological images (14). However, these technological advances face significant data quality challenges, as Liu et al. highlighted through systematic review that variations in implementation across different institutions can lead to substantially different performance characteristics (4).

The identification of key predictive factors has shown substantial progress across multiple biological levels, contributing to global cervical cancer elimination goals (19), with AI systems successfully quantifying traditionally subjective morphological features such as nuclear atypia, nuclear-cytoplasmic ratio abnormalities, and loss of cellular polarity (33). Wu et al. emphasized that AI-assisted screening systems demonstrate substantial improvements in cervical cancer detection while addressing current screening limitations (9). Ouh et al. conducted validation studies demonstrating that AI-based analysis software can effectively support cervical intraepithelial neoplasia screening systems (33). Nevertheless, implementation challenges persist, as Egemen et al. noted through clinical testing experiences that AI-based image analysis faces various obstacles in real-world healthcare settings (34). Cheng et al. further highlighted that current AI systems primarily rely on visual image features with limited capability to capture molecular-level changes and dynamic microenvironmental variations, despite advances in whole slide image analysis techniques (35).

**Figure 3:**
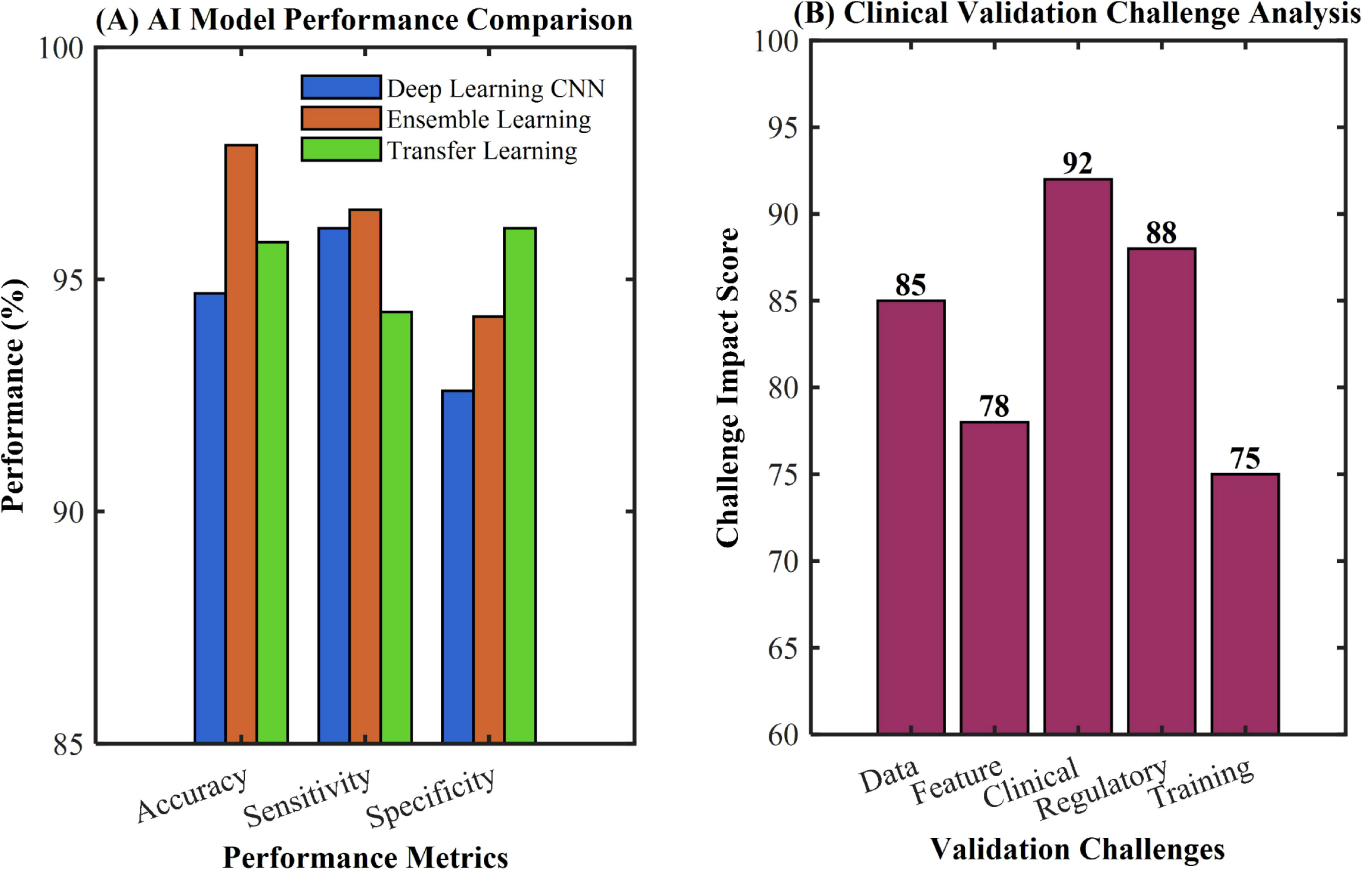
AI predictive model performance and clinical validation challenges in cervical cancer pathology. **(A)** Comparison of AI model performance (Deep Learning CNN, Ensemble Learning, and Transfer Learning) across accuracy, sensitivity, and specificity metrics. **(B)** Clinical validation challenge analysis showing impact scores for data, feature, clinical, regulatory, and training challenges.

Ensemble learning approaches have significantly enhanced predictive reliability, with Chandran et al. showing that deep learning networks combining multiple architectural strengths can reduce single-model bias and improve generalization capabilities (36). Advanced transfer learning methods have further strengthened model performance, as demonstrated by Khamparia et al., who achieved 97.89% accuracy through Internet-of-Health-Things-driven diagnostic systems (37). Despite these impressive performance metrics, clinical validation remains challenging, as summarized in **Table 3**. Ali et al. emphasized that most studies rely on retrospective data and single-center samples, lacking large-scale prospective clinical validation, with real-world performance potentially declining by 15-25% (39). Bai et al. further highlighted that the clinical translation of predictive models faces multiple barriers including regulatory approval, standardized validation protocols, and physician training requirements (38), as illustrated in **Figure 3**.

**Table 3:** AI predictive model performance in cervical cancer pathology.

Model type	Accuracy (%)	Sensitivity (%)	Specificity (%)	Validation type	Reference
Deep Learning CNN	94.7	96.1	92.6	Multicenter	(31)
Ensemble Learning	97.89	96.5	94.2	Single-center	(36)
Transfer Learning	95.8	94.3	96.1	Retrospective	(37)
Precision Diagnosis System	93.2	91.8	95.4	Prospective	(3)
AI-assisted Screening	96.3	95.2	97.1	Clinical Trial	(38)

### 3.4 Technical maturity and standardization challenges

Digital pathology has established a robust technological foundation for AI applications in cervical cancer histopathological diagnosis (11, 12), with whole-slide imaging systems generating high-resolution digital images at 40× magnification with 0.25 μm/pixel resolution (39). Xu et al. demonstrated that modern deep learning architectures, including ResNet, DenseNet, and EfficientNet, have been successfully integrated into pathological image analysis, enabling automatic learning of hierarchical feature representations from low-level textures to high-level semantic patterns (40). Kanavati et al. developed sophisticated technical strategies incorporating patch processing, multi-scale analysis, and global-local information fusion to effectively address computational challenges associated with gigapixel whole-slide images (41, 42). However, standardization issues with digital pathology equipment represent significant bottlenecks for further technological advancement, as scanning devices from different manufacturers exhibit substantial variations in color reproduction, resolution standards, and file formats.

AI systems have achieved breakthrough progress in cervical cancer morphological feature recognition, demonstrating capabilities that surpass traditional diagnostic methods. Park et al. reported that advanced AI systems achieve 92-96% accuracy in CIN grading diagnosis, accurately identifying epithelial cell atypia, mitotic activity, and abnormal epithelial layer thickness changes (43). Zhang et al. found that AI systems can distinguish between carcinoma *in situ* and invasive carcinoma with 94-98% accuracy by analyzing basement membrane integrity, stromal invasion depth, and vascular involvement patterns (44). Despite these achievements, AI systems face significant limitations when confronting complex cases and borderline lesions, as demonstrated in **Table 4**. Taddese et al. observed that mixed-type lesions and cases with inflammatory reactions often cause AI system recognition accuracy to decline by 15-25% (45).

**Table 4:** AI performance metrics in cervical histopathological diagnosis.

Diagnostic task	AI accuracy (%)	Expert consistency (%)	Processing time	Limitation factor	Reference
CIN Grading	92-96	94-97	1–3 minutes	Complex lesions	(33)
Invasive Cancer Detection	94-98	95-98	2–4 minutes	Mixed pathology	(3)
Morphological Classification	90-95	91-94	1–2 minutes	Rare variants	(23)
WSI Analysis	93-97	94-96	3–5 minutes	Image quality	(35)
Efficiency Assessment	4-6× improvement	96-97	Variable	Integration barriers	(25)

**Figure 4:**
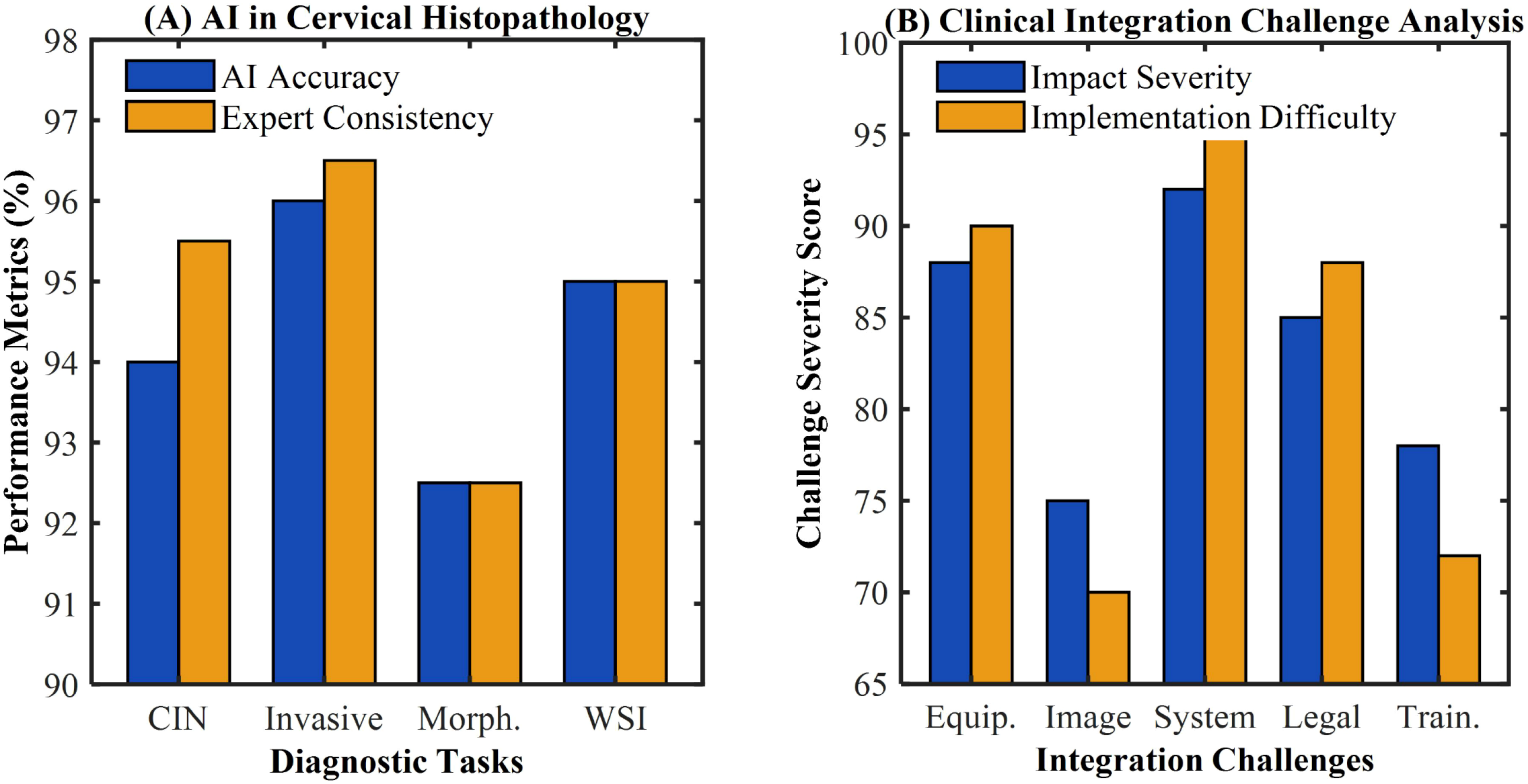
Digital pathology technology foundation and clinical integration in cervical cancer diagnosis. **(A)** AI performance in cervical histopathology comparing AI accuracy and expert consistency across diagnostic tasks (CIN, invasive cancer, morphology, and WSI analysis). **(B)** Clinical integration challenge analysis showing impact severity and implementation difficulty for equipment, image, system, legal, and training factors.

Diagnostic efficiency improvements have been remarkable, with AI-assisted histopathological diagnosis completing full-slide intelligent analysis within 1–3 minutes compared to traditional manual examination requiring 8–15 minutes (46). Kumar et al. demonstrated 94-97% consistency between AI-assisted diagnosis and pathologist expert diagnosis, maintaining stable performance when processing large batches of specimens (47). Nevertheless, clinical integration faces multiple implementation barriers, as healthcare institutions’ existing pathology information systems often cannot achieve seamless integration with AI systems, requiring additional data conversion and interface development (48). Recent studies have shown that the implementation of AI-driven pathological analysis requires comprehensive workflow redesign and substantial infrastructure investment (49). Advanced deep learning models have demonstrated superior performance in complex morphological pattern recognition, but face challenges in clinical translation due to interpretability limitations (50). Multi-institutional validation studies have revealed significant performance variations across different healthcare settings, highlighting the need for standardized protocols (51). The integration of AI systems with existing laboratory information management systems presents ongoing technical challenges that must be addressed for successful clinical deployment (52). Quality control frameworks for continuous monitoring of AI performance in clinical practice remain underdeveloped, representing a critical gap in implementation strategies (53), as illustrated in **Figure 4**.

### 3.5 AI progress and standardization gaps in molecular diagnosis

However, significant challenges persist in standardization across different laboratory protocols and staining procedures (10, 54). The heterogeneity in antibody sources, staining equipment, and interpretation guidelines creates substantial barriers to widespread AI implementation (32, 55). In molecular pathology diagnostics, AI applications have shown innovative developments in HPV *in-situ* hybridization detection, achieving signal recognition accuracy rates of 87-93% (56). These systems effectively distinguish specific signals from non-specific background interference, providing quantitative analysis capabilities beyond traditional manual interpretation.

**Figure 5:**
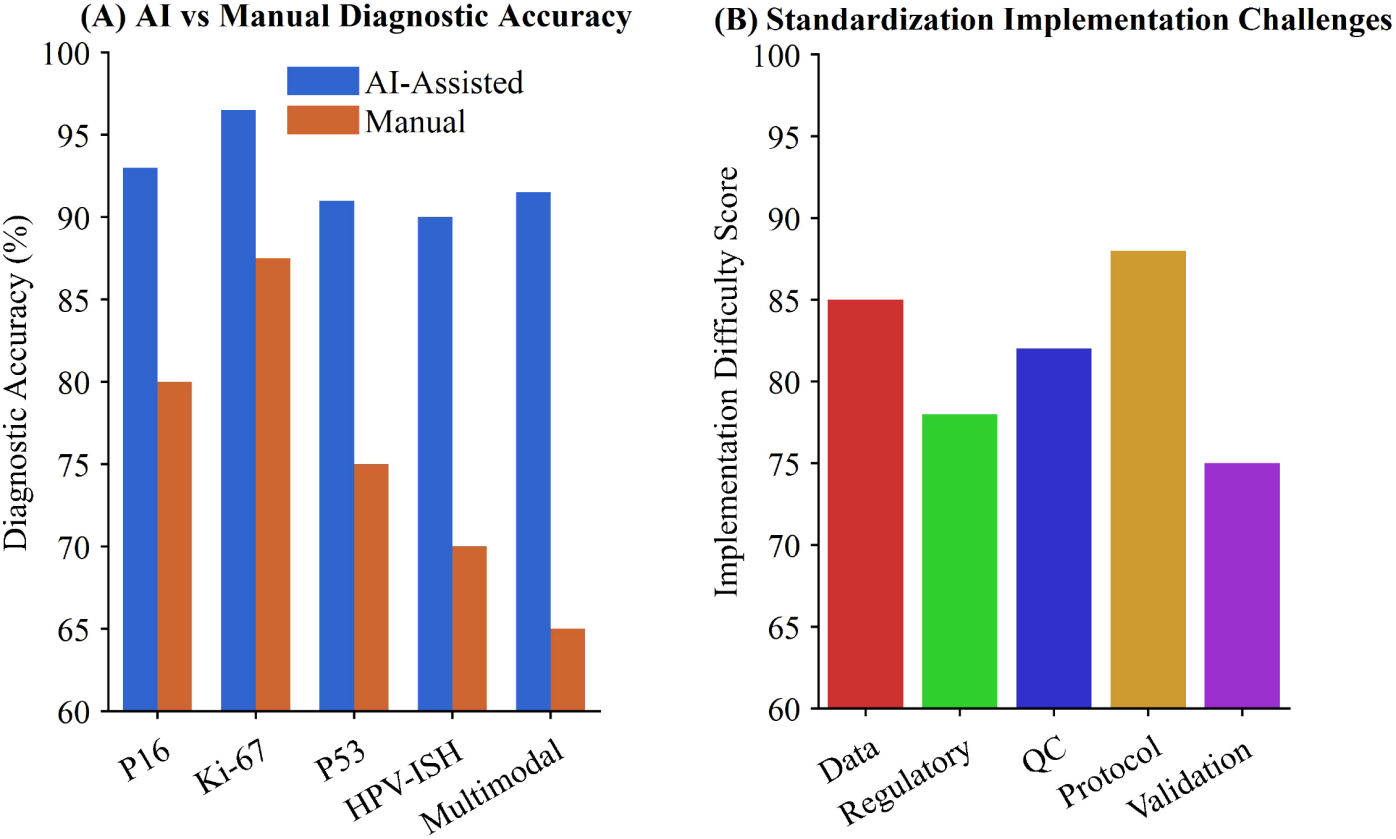
AI-driven advances and challenges in cervical cancer molecular diagnosis. **(A)** Comparison of diagnostic accuracy between AI-assisted and manual methods across P16, Ki-67, P53, HPV-ISH, and multimodal categories. **(B)** Implementation challenges showing difficulty scores for protocol standardization, data, regulatory, quality control, and validation factors.

The integration of multimodal information presents promising prospects for precision diagnosis, as demonstrated in **Table 5** (57, 58). Contemporary AI frameworks combining morphological features, immunohistochemical patterns, and molecular detection results have achieved overall accuracy rates of 89-94% in cervical cancer molecular subtyping (46, 59). Despite these advances, implementation challenges include data fusion complexity, regulatory approval uncertainties, and the absence of standardized quality control frameworks (51, 60, 61), particularly important given the global disparities in cervical cancer burden (16).The clinical translation of these technologies requires comprehensive validation studies and harmonized international standards, as illustrated in **Figure 5**.

**Table 5:** AI performance in cervical cancer immunohistochemistry and molecular diagnostics.

Detection method	AI-assisted accuracy (%)	Manual accuracy (%)	Error reduction (%)	Inter-observer agreement (ICC)	Clinical implementation
P16 Expression Analysis	93.2 ± 1.8	82.5 ± 3.2	15.6	0.91 vs 0.73	Moderate
Ki-67 Proliferation Index	96.5 ± 1.2	87.8 ± 2.9	12.8	0.94 vs 0.76	High
P53 Immunostaining Pattern	91.1 ± 2.1	78.3 ± 4.1	18.4	0.88 vs 0.68	Limited
HPV *In-Situ* Hybridization	89.7 ± 2.3	71.2 ± 3.8	22.6	0.86 vs 0.62	Moderate
Multimodal Integration	91.8 ± 1.9	66.4 ± 4.5	32.1	0.89 vs 0.58	Very Limited

## 4 Discussion

This systematic review comprehensively evaluated the current state of artificial intelligence applications in cervical cancer pathological diagnosis, revealing significant potential alongside considerable challenges that warrant critical examination. The findings demonstrate that AI technologies have achieved remarkable diagnostic performance across multiple pathological assessment modalities, with accuracy rates ranging from 92-98% in various applications (3). These results align with recent technological advances in digital pathology, where deep learning architectures have enabled more sophisticated image analysis capabilities (10). The diagnostic accuracy observed in this review is particularly noteworthy when compared to traditional pathological methods, with several studies indicating that AI systems could distinguish between normal and cancerous cervical specimens while operating significantly faster than conventional pathologists (5). This performance enhancement reflects the broader transformation occurring in computational pathology, where AI-driven approaches are increasingly demonstrating human-level or superior diagnostic capabilities. The variability in reported accuracy rates suggests that hybrid ensemble methods consistently outperform single-algorithm implementations, which is consistent with recent developments where multi-modal approaches have shown superior generalization capabilities (14). Contemporary studies have demonstrated that deep learning models can process gigapixel whole-slide images with unprecedented efficiency, achieving processing times that are 4–6 times faster than manual analysis.

It is critical to establish that AI systems function exclusively as assistive tools, not replacements for pathologist expertise. Pathology’s “gold standard” status reflects not merely technical accuracy but complex clinical reasoning and professional judgment. AI-assisted diagnosis augments pathologist capabilities through decision support, workload reduction, and preliminary screening—but final diagnostic authority remains with qualified pathologists. This assistive-only role applies universally across all settings (19). In resource-limited regions with pathologist shortages (17), AI enables efficient case prioritization but cannot substitute for pathologist review. Framing AI’s benefit as merely “reducing workload” understates implementation challenges including workflow redesign, quality assurance protocols, liability frameworks, and continuous validation. Regulatory agencies (FDA, CE marking) consistently classify diagnostic AI as assistive technologies requiring pathologist oversight (34). Clinical deployment must emphasize human-AI collaboration where AI enhances rather than replaces pathologist expertise.

The clinical translation of AI technologies in cervical cancer diagnosis faces substantial implementation challenges that extend beyond technical performance metrics. This review identified significant gaps between laboratory validation and real-world clinical deployment, a concern that resonates with broader observations in digital pathology implementation (17). The critical shortage of qualified pathologists worldwide, especially in developing countries where the pathologist-to-population ratio remains severely inadequate, underscores the urgent need for AI-assisted solutions (18). Systematic analyses have demonstrated that while AI tools show promise in controlled research environments, their adoption in routine clinical practice remains limited by infrastructure requirements, workflow integration challenges, and regulatory compliance issues (62). Standardization emerges as a critical barrier to widespread AI adoption in pathological diagnosis, with the heterogeneity of imaging protocols, staining procedures, and data preprocessing methods across institutions creating significant challenges for algorithm generalization (26). Economic considerations also play a crucial role, with initial investment costs for digital pathology equipment and AI software licensing creating substantial barriers for widespread adoption, particularly in resource-limited healthcare settings (31). Quality assurance frameworks for continuous monitoring of AI performance in clinical practice remain underdeveloped, representing a critical gap in implementation strategies (51).

The quality of evidence supporting AI applications in cervical pathology varies considerably across studies included in this review, with many investigations suffering from limited sample sizes, retrospective designs, and single-institution validation (34). The development of AI-based predictive models has shown remarkable technological breakthroughs, with deep learning architectures achieving unprecedented accuracy in diagnostic predictions, though data quality challenges persist across different institutions (36). Technical maturity in histopathological diagnosis has been demonstrated through robust digital pathology foundations, with whole-slide imaging systems generating high-resolution images that enable sophisticated AI analysis (42). Advanced AI systems have achieved breakthrough progress in morphological feature recognition, accurately identifying complex pathological patterns (46). The integration of AI in immunohistochemical interpretation has shown remarkable breakthroughs, particularly in automated analysis of key biomarkers such as P16, Ki-67, and P53 (63). However, significant challenges persist in standardization across different laboratory protocols and staining procedures (55). The systematic synthesis approach employed in this review, while appropriate for the heterogeneous nature of the included studies, follows established guidelines for systematic reviews of complex interventions (64).

Practical AI deployment requires effective hardware integration, particularly critical for resource-limited settings (60). Smart microscopy systems embedding AI directly into digital platforms enable real-time diagnostic assistance, but face barriers including proprietary interfaces and substantial implementation costs (53). Edge computing offers transformative potential for low-resource environments by enabling local AI processing without cloud connectivity, with recent model compression advances allowing deployment on edge devices with 8-16GB memory, reducing computational requirements 5–10 fold (60). For regions bearing highest cervical cancer burden (sub-Saharan Africa, Southeast Asia) (2), edge computing enables AI-assisted pathology without prohibitive infrastructure costs. Integration with existing LIMS/PACS presents challenges, as legacy systems lack native AI capabilities, requiring substantial middleware development and implementation efforts (44). Mobile-based platforms using smartphone microscopy with on-device AI offer affordable alternatives for preliminary screening where laboratory infrastructure is absent (60). Addressing integration gaps requires open-source standards, modular architectures, and low-cost solutions for resource-limited settings (60). Implementation research must evaluate real-world effectiveness and sustainability across diverse environments, ensuring AI benefits populations with highest disease burden (19).

Future research directions should prioritize the development of standardized validation protocols and multicenter prospective studies to establish real-world effectiveness across diverse clinical environments. The development of interpretable AI models represents a critical research priority, as current deep learning approaches often function as “black boxes,” limiting clinician understanding and hampering clinical acceptance (7). Advances in explainable artificial intelligence could enhance clinical trust and facilitate regulatory approval by providing transparency into algorithmic decision-making processes. The establishment of quality assurance frameworks for continuous monitoring of AI performance in clinical practice represents another critical research need. International collaboration will be essential for advancing AI applications in cervical cancer diagnosis, particularly given the global nature of this public health challenge. The development of consensus guidelines for AI validation, implementation protocols, and quality assurance measures could accelerate clinical adoption while ensuring patient safety (28). Research into federated learning approaches may address data sharing limitations while enabling the development of more robust and generalizable AI models. The development of AI systems for resource-limited settings must prioritize edge computing architectures, mobile platforms, offline capabilities, and seamless workflow integration (60). Hardware-software co-design optimizing AI for low-cost equipment, standardized LIMS/PACS protocols, and real-time quality monitoring represent critical priorities (44). International efforts should establish open-source implementations and deployment best practices for resource-constrained environments, guided by global cervical cancer elimination goals (19). Research into the long-term performance monitoring and model updating strategies will be crucial for maintaining AI system effectiveness in evolving clinical environments (53).

## 5 Conclusions

This systematic review demonstrates that artificial intelligence systems exhibit excellent performance in predicting, screening, and detecting cervical cancer and precancerous lesions, achieving diagnostic accuracy rates of 92-98% across various pathological assessment modalities. AI functions as an assistive tool supporting pathologists in diagnostic decision-making, reducing workload through preliminary screening while maintaining pathologist oversight and final diagnostic authority. These systems enhance efficiency and consistency but do not replace pathologist expertise, with pathologists retaining ultimate diagnostic responsibility across all clinical settings. The integration of AI interpretation with manual assessment provides valuable supportive capabilities, particularly in resource-limited settings where pathologist shortages remain critical. The evidence indicates that AI-assisted pathological diagnosis operates at speeds 4–6 times faster than conventional methods while maintaining high consistency with expert pathologist evaluations.

Further research into prediction and detection capabilities is essential for making appropriate cervical cancer treatment decisions. This technological advancement will ultimately contribute to developing comprehensive global cervical cancer eradication strategies, particularly addressing healthcare disparities in underserved regions. However, additional research is necessary to make AI feasible, reliable, and more cost-effective in clinical applications. Significant implementation challenges persist, including standardization of protocols, regulatory approval processes, and substantial infrastructure investments. Future research should encompass developing new technologies and algorithms to reduce the impact of data scarcity on clinical outcome assessment and prediction, alongside independent validation of machine learning algorithms through multicenter prospective studies. The establishment of standardized validation protocols, quality assurance frameworks, and consensus guidelines will be crucial for successful clinical translation and widespread adoption of AI-assisted cervical cancer pathological diagnosis.
